# Antibiotic resistance profiles and co-occurrence of ESBL and AmpC β-lactamase genes in ESBL-producing Gram-negative bacteria of clinical origin

**DOI:** 10.1186/s12866-026-05129-x

**Published:** 2026-05-27

**Authors:** Abimbola O. Adekanmbi, Lateef T. Ishola, Esther B. Ogunsakin, Temitope A. Ajamu, Fareedah T. Lawal

**Affiliations:** 1 Environmental Microbiology and Biotechnology Laboratory, Department of Microbiology, University of Ibadan, Ibadan, Oyo state Nigeria; 2 Molecular Biology and Biotechnology Laboratory, Department of Microbiology, University of Ibadan, Ibadan, Oyo state Nigeria; 3 Department of Science Laboratory Technology, Osun state Polytechnic, Iree, Osun state Nigeria; 4 Teesside University, Southfield, Middlesbrough, UK

**Keywords:** Clinical bacteria, ESBL-producing bacteria, Teaching Hospital, ESBL genes, AmpC β-Lactamase genes, Hypermucoviscous *Klebsiella pneumoniae*, Biofilm production, Multidrug-resistant (MDR)

## Abstract

The hospital setting provides a critical environment for understanding the public health implications of antibiotic resistance, given the diversity of clinical samples and bacteria encountered. This study investigated the resistotyping and detection of ESBL and AmpC β-lactamases in clinical Gram-negative bacteria. Bacteria were obtained from the laboratory benches of a Teaching Hospital in Nigeria for three months, and identified. Antibiotic susceptibility was carried out using the disc diffusion method, while detection of ESBL production was done using the Double Disc Synergy Test (DDST). Biofilm formation by the isolates was detected using the Tissue Culture Plate method. Screening for hypermucoviscosity in *Klebsiella pneumoniae* isolates was done using the string test. Genotyping of the ESBL-producing isolates for ESBL and AmpC β-lactamases was done using primer-specific PCR. Seventy-two isolates [(*Klebsiella* (38), *Pseudomonas* (14), *Escherichia* (12), *Enterobacter* (5), *Hafnia* (1), *Acinetobacter* (1) and *Morganella* (1)] were identified. The resistance of the isolates to the antibiotics was: amoxicillin-clavulanate (69.4%), cefepime (59.7%), ceftriaxone and azithromycin (56.9% each), aztreonam 54.2%, gentamicin (52.8%), ampicillin-sulbactam (48.6%), ciprofloxacin (45.8), ceftazidime (38.9%), cefoxitin (36.1%) and cefuroxime (19.4%). Fifty-three isolates (73.6%) had multidrug-resistant phenotypes and 21 were ESBL producers. Thirty-five of the total isolates were biofilm producers, while 21.1% of the *Klebsiella pneumoniae* were hypermucoviscous. The frequency of genes in the 21 ESBL producers was *bla*_TEM_ (35.3%), *bla*_CTX−M_ (29.4%), *bla*_SHV_ (23.5%) and *bla*_CMY_ (11.8%). The occurrence of MDR and ESBL-producing bacteria from clinics raises alarm on the need to further educate people on the consequences of indiscriminate usage of antibiotics without prescription.

## Introduction

The prevalence of antibiotic resistance remains a significant global health challenge, contributing to increased morbidity and mortality worldwide [1]. Gram-negative bacteria, including *Enterobacter aerogenes*, *Pseudomonas aeruginosa*, *Klebsiella pneumoniae*, *Acinetobacter baumannii*, and *Escherichia coli*, are among the primary pathogens implicated in both hospital-associated (nosocomial) and community-acquired infections globally [1]. These pathogens have developed diverse mechanisms to evade the effects of multiple antimicrobial agents, including aminoglycosides, fluoroquinolones, sulfonamides, and β-lactam antibiotics.

Beta-lactam antibiotics, including penicillins, cephalosporins, monobactams, and carbapenems, represent the most commonly employed agents in the treatment of infections caused by multidrug-resistant Gram-negative bacteria [2]. These antibiotics exert their bactericidal effects by targeting proteins essential for bacterial cell wall synthesis. Penicillin-binding proteins (PBPs), which catalyze the cross-linking of peptide chains during peptidoglycan biosynthesis, are the primary targets of β-lactams. By covalently binding to PBPs, β-lactam antibiotics inhibit peptidoglycan assembly, resulting in compromised cell wall integrity, bacterial lysis, and ultimately cell death [3].

Bacterial resistance to β-lactam antibiotics is escalating at an alarming pace. The primary mechanism of resistance in Gram-negative bacteria involves the production of β-lactamase enzymes [4]. Among the more than 1,000 known β-lactamases, Extended Spectrum β-lactamases (ESBLs) and class C β-lactamases (AmpC) are the most commonly encountered in Gram-negative bacteria [5]. ESBLs, typically plasmid-mediated, represent a diverse group of enzymes that confer resistance to a broad spectrum of frequently used β-lactam antibiotics [6]. These enzymes can hydrolyze various β-lactams, including penicillins and cephalosporins, and often facilitate co-resistance to other antibiotic classes such as fluoroquinolones and aminoglycosides.

The incidence of infections caused by ESBL-producing Gram-negative bacteria is rising globally, affecting both healthcare and community settings and presenting significant therapeutic challenges [7]. Similarly, AmpC β-lactamases confer resistance to β-lactam antibiotics and may be either plasmid- or chromosome-encoded. Epidemiological studies have shown that AmpC β-lactamase-producing bacteria are frequently isolated from hospitalized patients after several days of admission, with affected individuals often experiencing prolonged hospital stays [8].

The simultaneous presence of AmpC and ESBLs in Gram-negative bacteria has been frequently documented, with AmpC enzymes often masking the detection of ESBLs, as noted by Salvia et al. [9]. Consequently, Gram-negative bacteria producing these β-lactamases frequently evade detection, contributing to numerous nosocomial outbreaks. This situation is further exacerbated by inappropriate, indiscriminate, or delayed antimicrobial therapy [10]. Without precise laboratory identification and reporting of these resistance genes, treatment of infections caused by Gram-negative bacteria may remain suboptimal. In Nigeria, reports on the co-existence of ESBL and AmpC β-lactamases in human clinical samples are limited, highlighting the importance of this study. Therefore, this study offers a comprehensive analysis of the genetic diversity of Extended Spectrum and AmpC β-lactamases in bacteria isolated from human clinical samples over a specified period of time, along with their resistance profiles against commonly-used antibiotics.

## Materials and methods

### Ethical approval

Ethical approval for this study was sought from the Research Ethics Committee of Hospitals Management Board, Oyo State Ministry of Health and was granted under the number: NHREC/OYOSHRIEC/10/11/22.

### Bacteria used for the study

Clinical bacterial isolates obtained from urine, blood, sputum, wound swabs, tracheal aspirates, and amniotic fluid were collected from laboratory work benches in the Department of Microbiology and Parasitology at University College Hospital (UCH), Ibadan, Oyo State. University College Hospital is a leading tertiary healthcare institution in Ibadan, located on latitude 7.4019° N and longitude 3.9021° E. The isolates were obtained from the samples using the streak plate method on different culture media. Collection of isolates was done weekly for a period of three months. Approval was sought from the Department before access was granted to the laboratory benches for isolate collection.

### Identification of Isolates

The isolates were identified using the Microbact™ Gram Negative System Identification Kit (Thermo Scientific-Oxoid, UK) that consists of dehydrated substrates for 24 different biochemical tests placed in the wells of a microtitre tray. The inoculum was prepared by picking one or two colonies of the bacteria and introducing into sterile normal saline (3mL) to form a suspension. The saline suspension of each test organism was added to each of the 24 wells and appropriate procedures were followed for each well. Suitable reagents were added after overnight incubation at 35 ± 2 °C, and colour changes of the different tests were observed and recorded. The results were transcribed into a code and organisms were identified by the use of a computer-based profile register.

### Antibiotic susceptibility testing, detection of ESBL and AmpC β-lactamase production

The antibiotic susceptibility profiles of the bacterial isolates were determined using the standard disc diffusion method [11]. The antibiotics evaluated included ciprofloxacin (5 µg), aztreonam (30 µg), gentamicin (10 µg), ceftazidime (30 µg), cefoxitin (30 µg), azithromycin (15 µg), amoxicillin-clavulanate (30 µg), ampicillin-sulbactam (20 µg), cefepime (30 µg), ceftriaxone (30 µg), erythromycin (15 µg), and cefuroxime (30 µg) (Oxoid, United Kingdom). Each isolate was subcultured for 18–24 h, and one or two colonies were inoculated in sterile normal saline to achieve a turbidity equivalent to 0.5 McFarland standard. The standardized inoculum of each bacterium was uniformly spread onto Mueller-Hinton agar plates using sterile swab sticks. Antibiotic discs were aseptically placed on the agar surface using sterile forceps. The plates were then incubated at 35 ± 2 °C for 18–24 h. Following incubation, the diameters of the zones of inhibition were measured in millimeters and interpreted according to CLSI criteria [12].

Extended Spectrum β-lactamase (ESBL) production was screened phenotypically using the Double Disc Synergy Test (DDST), following CLSI recommendations [12]. Isolates exhibiting resistance to three or more antibiotic classes were categorized as multidrug-resistant (MDR) based on the criteria established by Magiorakos et al. [13]. Phenotypic detection of AmpC β-lactamase in the isolates was done using the Cefoxitin-Cloxacillin Double Disc Synergy (CC-DDS) test. Isolates phenotypically identified as ESBL producers were subsequently selected for the PCR detection of ESBL and AmpC β-lactamase genes.

### Screening of *Klebsiella pneumoniae* for Hypermucoviscosity

Hypermucoviscosity (HMV) phenotypes in the *Klebsiella pneumoniae* isolates obtained were determined using the string test, as previously described by Fang et al. [14]. Fresh overnight cultures grown on blood agar at 37 °C were used for the assay. A sterile inoculating loop was used to gently touch a single colony, which was then lifted vertically from the agar surface. The formation of a mucoviscous string was visually evaluated. An isolate was classified as HMV-positive if the colony produced a string measuring ≥ 5 mm in length upon lifting. Isolates producing strings < 5 mm or showing no visible string formation were considered HMV-negative. Each test was performed in triplicate under aseptic conditions to ensure reproducibility, and string lengths were measured using a standard laboratory ruler.

### Biofilm Formation Assay

The ability to form biofilms by the bacteria was determined using the Tissue Culture Plate (TCP) method, as described by Christensen et al. [15]. All experiments were conducted in triplicates. The bacteria were classified as non-biofilm producer; weak biofilm producer; moderate biofilm producer; and strong biofilm producer according to the interpretation method of Stepanović et al. [16].

### DNA extraction and PCR amplification of ESBL and AmpC β-lactamase genes

Genomic DNA of the bacterial isolates showing a positive phenotypic ESBL test was extracted using a Zymo Research Bacterial DNA MiniPrepTM Kit (Zymo Research Corporation, USA) following the manufacturer’s instructions. The eluted DNA was kept at -20 °C prior to use. A multiplex PCR was used to amplify the *bla*_SHV_ and *bla*_TEM_ genes in the ESBL-producing isolates [17]. The PCR conditions are as given: initial denaturation step at 94 °C for 5 min, denaturation at 94 °C for 30 s, primer annealing at 50 °C for 30 s, extension at 72 °C for 90 Sect. (30 cycles), and final extension at 72 °C for 10 min. The *bla*_CTX−M_ was separately amplified using a monoplex PCR [18]. with an initial denaturation of 94 °C for 5 min, denaturation at 94 °C for 30 s, primer annealing at 56 °C for 1 min, extension at 72 °C for 60 Sect. (30 cycles) and final extension at 72 °C for 10 min. *bla*_CMY_ was amplified using a monoplex PCR protocol as reported by Mandakini et al. [19]. Amplicons were separated on 1% agarose gel using electrophoresis. The primer sequences of the target genes are shown in Table 1. A multidrug-resistant *E. coli* ALC08 isolated from abattoir leachate, carrying *bla*_CTX−M_, *bla*_SHV_ and *bla*_TEM_ as reported by Adekanmbi et al. [20]. was used as the positive control.

**Table 1:** Oligonucleotide primers used in this study

Target gene	Primer sequence	Amplicon size (bp)	Reference
*bla*_TEM_	F-GAGTATTCAACATTTTCGT R-ACCAATGCTTAATCAGTGA	857	[17]
*bla*_SHV_	F-TCGCCTGTGTATTATCTCCC R-CGCAGATAAATCACCACAATG	768	[17]
*bla*_CTX−M_	F-TTTGCGATGTGCAGTACCAGTAA R-CGATATCGTTGGTGGTGCCATA	543	[18]
*bla*_CMY_	F-TGGCCAGAACTGACAGGCAAA R- TTTCTCCTGAACGTGGCTGGC	462	[19]

## Results

### Distribution of isolates according to sample source

Seventy-two clinical Gram-negative bacteria were obtained in total within the duration of study and the distribution of the isolates from the various clinical samples is presented in Table 2. The highest number of clinical Gram-negative bacteria was obtained from urine 28 (38.9%). This was followed in that order by blood 24 (33.3%), wound 8 (11.1%), sputum 7 (9.7%), amniotic fluid 4 (5.6%) while the least prevalence of clinical isolates was from tracheal aspirate 1 (1.4%).

**Table 2:** Distribution of clinical Gram-negative isolates according to samples

Sample	No. of Isolates obtained	Percentage (%)
Urine	28	38.9
Blood	24	33.3
Wound	8	11.1
Sputum	7	9.7
Amniotic fluid	4	5.6
Tracheal aspirate	1	1.4
	72	100

### Frequency of clinical Gram-negative bacteria recovered from the clinical sources according to species

The frequency of clinical Gram-negative bacterial species obtained in this study is shown in Table 3. Thirty eight isolates of the total isolates obtained were identified as *Klebsiella pneumoniae* with a frequency of 52.8%, 14 (19.4%) were identified as *Pseudomonas aeruginosa*, *Escherichia coli* constituted 12 (16.7%), followed by *Enterobacter cloacae* with five isolates (6.9%). *Hafnia alvei*,* Acinetobacter baumannii* and *Morganella morganii* had one isolate each.

**Table 3:** Frequency of clinical Gram negative bacteria obtained

Bacterial group	No. of Isolates obtained	Percentage (%)
*Klebsiella pneumoniae*	38	52.8
*Pseudomonas aeruginosa*	14	19.4
*Escherichia coli*	12	16.7
*Enterobacter cloacae*	5	6.9
*Hafnia alvei*	1	1.4
*Acinetobacter baumannii* *Morganella morganii*	1 1	1.4 1.4
Total	72	100

### Susceptibility to antibiotics

**Fig. 1:**
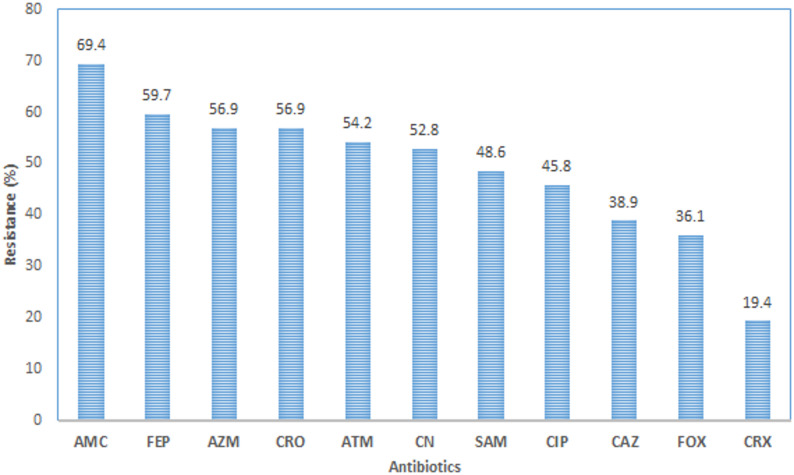
Resistance of clinical Gram negative bacteria to a selected panel of antibiotics. KEY: AMC = Amoxicillin-clavulanate; FEP = Cefepime; AZM = Azithromycin; CRO = Ceftriaxone; ATM = Aztreonam; CN = Gentamicin; SAM = Ampicillin-sulbactam; CIP = Ciprofloxacin; CAZ = Ceftazidime; FOX = Cefoxitin; CRX = Cefuroxime

The resistance of the 72 isolates obtained in this study to commonly-used antibiotics is presented in Fig. 1. The highest resistance was observed to amoxicillin-clavulanate (69.4%). The resistance to the other antibiotics was: cefepime (59.7%), ceftriaxone (56.9%), azithromycin (56.9%), aztreonam 54.2%, gentamicin (52.8%), ampicillin-sulbactam (48.6%), ciprofloxacin (45.8), ceftazidime (38.9%), cefoxitin (36.1%) and cefuroxime (19.4%). Fifty-three isolates representing 73.6% of the total isolates were observed to have multidrug resistant phenotypes (resisted ≤ 3 classes of antibiotics). The multiple antibiotic resistance index (MARI) of the 72 isolates ranged from 0 to 1.00 with two isolates showing resistance to the 11 tested antibiotics and two observed to be sensitive to all the antibiotics tested.

### Phenotypic production of ESBL by the isolates

Of the 72 isolates screened for ESBL production, 21 isolates were confirmed to produce ESBL, giving an overall prevalence of 29.2%. The ESBL-producing isolates were *Klebsiella pneumoniae* (9), *E. coli* (6), *Enterobacter cloacae* (3), *Pseudomonas aeruginosa* (1), *Acinetobacter baumannii* (1) and *Hafnia alvei* (1) as shown in Table 4.

**Table 4:** Frequency of ESBL producing-bacteria from the clinical samples

Bacterial group	No. of isolates obtained	No. of ESBL-producing bacteria
*Acinetobacter baumannii*	1	1
*Hafnia alvei*	1	1
*Enterobacter cloacae*	5	3
*Escherichia coli*	12	6
*Klebsiella pneumoniae*	38	9
*Pseudomonas aeruginosa*	14	1
*Morganella morganii*	1	0
Total Number	72	21/72 (29.2%)

### Hypermucoviscosity in *K. pneumoniae* isolates

Among the 38 *K. pneumoniae* isolates obtained, eight isolates (21.1%) formed strings of > 5 mm, were classified positive for the string test, and termed hypermucoviscous *K. pneumoniae* (HMKP). All the HMKP isolates had multidrug-resistant (MDR) phenotypes.

### Biofilm Production

**Fig. 2:**
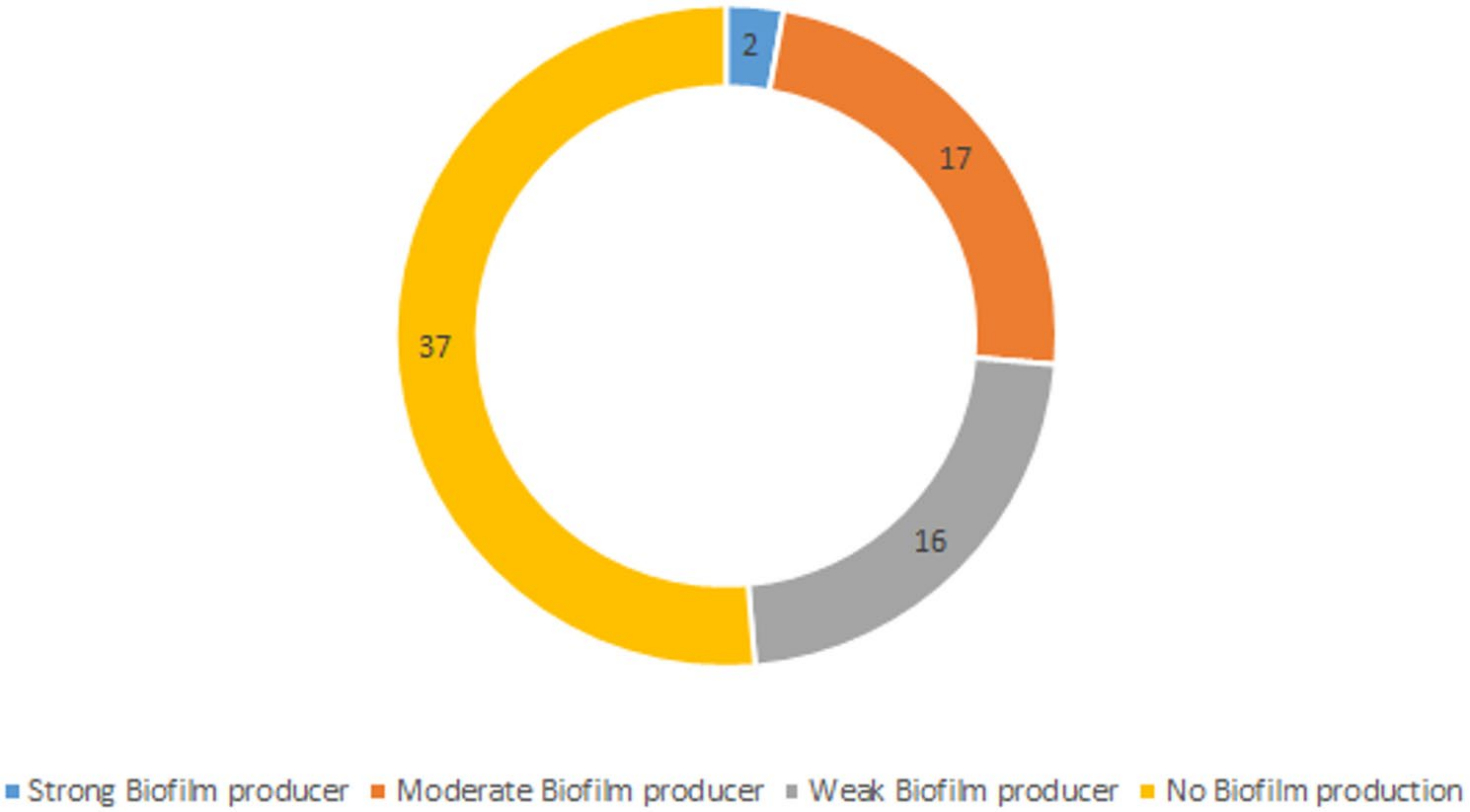
Biofilm formation in the isolates obtained from clinical samples

The biofilm production assay showed that 35 (48.6%) of the isolates demonstrated different degree of biofilm formation and 37 (51.4%) were non-biofilm producers. Moderate biofilm production was observed in 17 isolates, followed by weak biofilm production, which was observed in 16 isolates. Two isolates were termed strong biofilm producers according to the assay (Fig. 2).

### Distribution of ESBL and AmpC β-lactamase genes in the clinical isolates

**Fig. 3:**
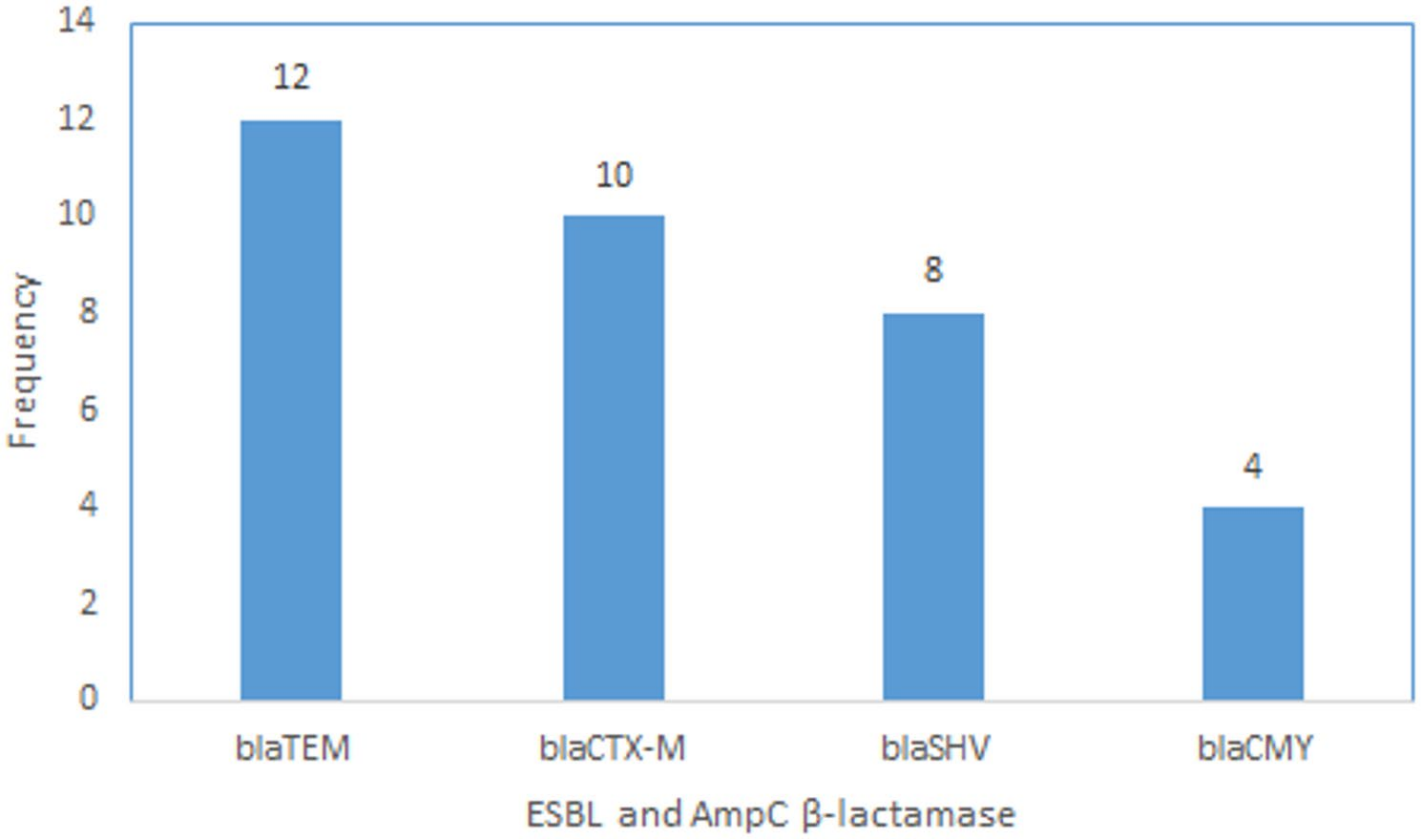
Presence of ESBL and AmpC β-lactamase genes among Clinical Isolates

The distribution of β-lactamase genes among the 21 ESBL-producing clinical Gram negative bacteria obtained is summarized in Table 5, while the frequency of ESBL and AmpC β-lactamases in the same set of isolates is illustrated in Fig. 3. The most prevalent ESBL gene among the ESBL positive isolates was *bla*_TEM_ which was detected in 12 (35.3%) of the isolates. This was followed by *bla*_CTX−M_ 10 (29.4%) and *bla*_SHV_ 8 (23.5%). The only AmpC β-lactamase targeted in this study (*bla*_CMY_) was detected in 11.8% of the total 21 ESBL-producing isolates. *E. coli* 192 isolated from blood was the only organism in this study that carried all the ESBL and AmpC β-lactamase targeted, while *K. pneumoniae* 50 carried all the three ESBL genes. No ESBL or AmpC β-lactamase was detected in *E. coli* 190, *A. baumannii* 56 and *H. alvei* respectively.

**Table 5:** Distribution of ESBL and AmpC β-lactamase genes in the ESBL-producing Gram-negative bacteria from clinical samples

Isolate	Source	Resistance gene profile
*A. baumannii* 56	Urine	None
*E. aerogenes*196	Wound	*bla*_CTX−M_, *bla*_TEM_
*E. cloacae* 002	Wound	*bla*_CTX−M_, *bla*_CMY_
*E. cloacae* 195	Wound	*bla*_CTX−M_, *bla*_CMY_
*E. coli* 12	Urine	*bla*_TEM_, *bla*_CMY_
*E. coli* 13	Tracheal aspirate	*bla*_CTX−M,_ *bla*_TEM_, *bla*_SHV_
*E. coli* 17	Blood	*bla*_CTX−M,_ *bla*_SHV_
*E. coli* 190	Amniotic fluid	None
*E. coli* 191	Urine	*bla*_TEM_
*E. coli* 192	Blood	*bla*_CTX−M_, *bla*_TEM_, *bla*_SHV_, *bla*_CMY_
*H. alvei*	Wound	None
*K. pneumoniae* 0001	Sputum	*bla*_TEM,_ *bla*_SHV_
*K. pneumoniae* 129	Urine	*bla*_CTX−M,_ *bla*_SHV_
*K. pneumoniae* 131	Urine	*bla*_TEM_
*K. pneumoniae* 134	Urine	*bla*_CTX−M,_ *bla*_TEM_
*K. pneumoniae* 193	Sputum	*bla*_TEM,_ *bla*_SHV_
*K. pneumoniae* 24	Sputum	*bla*_CTX−M_
*K. pneumoniae* 50	Blood	*bla*_CTX−M,_ *bla*_TEM_, *bla*_SHV_
*K. pneumoniae* 53	Blood	*bl*a_SHV_
*K. pneumoniae* 67	Urine	*bla*_TEM_
*Pseudomonas* spp. 94	Urine	*bla*_TEM_

## Discussion

Antimicrobial resistance is increasing at a pace that far outstrips the development and approval of new therapeutic agents, posing a significant threat to global public health. The widespread availability and inappropriate use of antibiotics have accelerated the emergence and dissemination of resistant bacterial strains, leading to a marked rise in infections that are difficult to treat in healthcare settings. Among the various resistance mechanisms identified in Gram-negative bacteria, the production of Extended Spectrum β-lactamase (ESBL) is particularly concerning and has been reported extensively worldwide [21]. ESBL-producing organisms are capable of hydrolyzing a wide range of β-lactam antibiotics, thereby limiting treatment options and contributing to higher morbidity and mortality rates. According to Arena et al. [22], Gram-negative bacteria are frequently isolated from diverse clinical specimens, including wound swabs, blood, sputum, amniotic fluid, tracheal aspirates, and urine, representing the most prevalent group of pathogens encountered in most Clinical Microbiology Laboratories.

In this present study, seventy-two Gram-negative bacterial isolates were recovered from diverse clinical specimens collected at a Tertiary Healthcare facility located in Southwestern Nigeria. *Klebsiella pneumoniae* represented the most frequently isolated clinical species, accounting for 52.8% of the total isolates, followed by *Pseudomonas aeruginosa* (19.4%). These findings are consistent with previous reports by Navon-Venezia et al. [23]. and Kibuchi et al. [24]., who similarly identified *K. pneumoniae* as the predominant Gram-negative pathogen in samples obtained from the clinical settings.

*Klebsiella pneumoniae* continues to pose a significant public health challenge, being implicated in a broad spectrum of infections such as respiratory tract infections, urinary tract infections (UTIs), and septicemia [25]. Notably, Jalil et al. [26]. also reported *K. pneumoniae* as the most prevalent bacterium in urine-derived samples. Analysis of specimen origin revealed that blood samples exhibited the highest prevalence of Gram-negative bacteria (42.1%), followed by urine samples (31.6%). This distribution pattern aligns with observations made by Salmanov et al. [27]., who reported a higher incidence of Gram-negative organisms in blood (61.2%) and urine (16.4%). The observed differences in percentage distributions across studies may be attributed to variations in sample size, study duration, and methodological approaches.

In the current study, *Klebsiella pneumoniae* isolates exhibiting the hypermucoviscous (HMV) phenotype accounted for 21.1% of the total *K. pneumoniae* isolates. This prevalence exceeds that reported by Wang et al. [28]., who documented a lower proportion of HMV *K. pneumoniae* among carbapenem-resistant clinical isolates in a Tertiary care hospital in China. Nonetheless, the prevalence observed in this study remains substantially lower than the 47% HMV rate reported by Umar et al. [29]. in a separate investigation of clinical *K. pneumoniae* isolates. Notably, consistent with the findings of Umar et al. [29]., all HMV *K. pneumoniae* isolates in the present study displayed multidrug-resistant (MDR) phenotypes. This observation supports a positive association between the hypermucoviscous phenotype and multidrug resistance, further underscoring the clinical significance of HMV *K. pneumoniae* in antimicrobial resistance surveillance.

Although previous studies have established a strong association between Extended Spectrum β-lactamase (ESBL) production and biofilm formation in bacterial pathogens, particularly *Klebsiella pneumoniae* [30, 31], the current study observed biofilm formation exclusively among non ESBL-producing isolates. This finding diverges from earlier reports and may be attributable to strain-specific genetic factors or environmental conditions influencing biofilm production. In this study, 48.6% of *K. pneumoniae* isolates demonstrated biofilm-forming capacity, reinforcing the role of biofilm as a critical virulence determinant in the pathogenesis of this organism. This proportion is comparable to a study conducted in Nepal, where 73.3% of *K. pneumoniae* isolates were reported to form biofilms [32]. Among the biofilm-producing isolates in this present study, moderate (23.6%) and weak (22.2%) producers predominated, while only 2.8% of isolates were classified as strong biofilm formers. A similar trend was reported in a study on methicillin-resistant *Staphylococcus aureus* (MRSA) in China, where 53.2% of isolates exhibited biofilm-forming ability, predominantly of weak to moderate strength [33].

Furthermore, comparable findings were noted in a study of uropathogenic *Escherichia coli* (UPEC) from Egyptian hospitals, in which only 8% of isolates were non-biofilm producers, while 32%, 29%, and 31% of isolates were categorized as weak, moderate, and strong biofilm producers, respectively [34]. Conversely, Nirwati et al. [35]. reported a significantly higher rate of biofilm formation (85.6%) among *K. pneumoniae* isolates in Indonesia, including 33% strong, 52.1% moderate, and 8.5% weak biofilm producers. The ability of *K. pneumoniae* to form biofilm has been strongly implicated in the development of multidrug resistance and has been associated with the dissemination of various β-lactamase gene mechanisms [36], further underscoring the clinical importance of biofilm surveillance in antimicrobial resistance management.

The present study revealed a relatively high level of resistance among *Klebsiella pneumoniae* and other Gram-negative isolates to multiple antibiotic classes. These findings are consistent with previous reports, including that of Fang et al. [37]., who documented similar resistance patterns in Gram-negative pathogens isolated from the hospital settings. Comparable trends have also been observed in studies from Ethiopia, Uganda, and Ghana [38–40]. Reduced susceptibility was observed to the antibiotics tested in this study. This trend mirrors those reported in studies conducted in Ethiopia [41], Tanzania [42], Iran [43], and Sierra Leone [44].

Extended Spectrum β-lactamase (ESBL)-producing Gram-negative bacteria were isolated from a variety of clinical specimens, including urine, blood, amniotic fluid, tracheal aspirates, sputum, and wound swabs, consistent with reports by Aibinu et al. [45]., Chatterjee et al. [46]., Ozçakar et al. [47]. and Dalela et al. [48]. In this study, ESBL-producing organisms were most commonly isolated from urine (38.1%), followed by blood and wound samples (each 19.0%), likely reflecting the higher number of urine and blood specimens processed. These findings align with previous data from Ghana (66.7%) [38] and India (52.3%) [49]. However, in contrast, a study from Adama, Ethiopia identified blood as the predominant source of ESBL-producing organisms [40].

The overall prevalence of ESBL production among clinical isolates in this study was 29.2%, which is lower than that reported in studies from Addis Ababa, Ethiopia (57.7%) [50], Northwest Nigeria (58.0%) [51], and Southwest Uganda (89%) [52]. Such variability in ESBL prevalence may be attributable to differences in study populations, specimen types, sample sizes, antimicrobial stewardship policies, and geographical and temporal factors [53]. Nevertheless, the prevalence reported here exceeds that observed in other regions, including Ethiopia (25.0%) [40], Nepal (26.8%) [54], and Italy (6.3%) [55].

Within Nigeria, previous studies have documented varying ESBL prevalence: 44.6% in Enugu [56], 6.7% in Ebonyi [56], 20% in Lagos [45], 10.3% in Kano [57], and 36.6% in Benin [58]. At the global level, Castanheira et al. [59]. reported prevalence rates of 32.7% in Latin America, 26.6% in the Asia-Pacific region, 19.2% in Europe, and 16.4% in North America. The comparatively higher prevalence observed in this study may reflect less stringent infection control practices and broader, often unregulated, antibiotic use in the study setting, as previously suggested [60, 61].

Globally, the frequency of healthcare-associated infections caused by ESBL-producing pathogens continues to rise [62]. In the current study, 21 isolates were phenotypically confirmed as ESBL producers by PCR, of which 85.7% (*n* = 18) harbored at least one ESBL-encoding gene. The remaining 14.3% of phenotypic ESBL-positive strains lacked *bla*_CTX−M_, *bla*_TEM_, and *bla*_SHV_, suggesting the potential presence of other ESBL determinants not targeted in this study.

Among the ESBL gene-positive isolates, *K. pneumoniae* accounted for the highest proportion (42.9%), followed by *Escherichia coli* (28.6%). These findings are consistent with those of Moosavian and Deiham [63], who reported a higher prevalence of ESBL genes in *Klebsiella* spp. (79.5%). In terms of specific genes, *bla*_TEM_ was the most frequently detected (35.3%), followed by *bla*_CTX−M_ (29.4%) and *bla*_SHV_ (23.5%). The predominance of *bla*_TEM_, which confers resistance to penicillins and first-generation cephalosporins, is consistent with findings from Turkey and Italy, where its prevalence in hospital settings ranged between 52.7% and 56.4% [64, 65]. These results contrast with studies by Coque et al. [66]. and Ben-Ami et al. [67]., which identified *bla*_CTX−M_ as the most widespread ESBL gene globally.

Co-carriage of multiple ESBL genes and *bla*_CMY,_ an AmpC beta-lactamase was also observed in this study. Of the 18 ESBL gene-positive isolates, 12 (66.7%) harbored combinations of *bla*_TEM_, *bla*_CTX−M_, and/or *bla*_SHV_, in addition to four isolates [*E. cloacae* (2) and *E. coli* (2)] carrying *bla*_CMY_, which offers resistance to some cephalosporins and penicillins. The spread of these genes, often via plasmids, poses a challenge in treating infections, especially in patients with compromised immune systems. The co-occurrence of ESBL and AmpC beta-lactamase in isolates of clinical origin has been reported earlier in previous studies [68]. This finding is consistent with reports from Egypt [69, 70], Burkina Faso, Qatar, and Iran [71–73], and supports earlier observations of high rates of gene co-carriage, particularly in resource-limited settings. In southern Nigeria, *bla*_TEM_ has been reported as the predominant ESBL variant [74], while *bla*_SHV_ predominates in northern regions, as demonstrated by Mohamed et al. [75]. The co-existence of multiple resistance genes on mobile genetic elements such as plasmids and transposons may explain the high rates of multidrug resistance observed in ESBL-producing organisms [76], emphasizing the need for continuous molecular surveillance and strengthened antimicrobial stewardship interventions.

## Limitation to the study

The limitation of this study include the relatively small number of clinical isolates and the restricted sampling duration. These constraints were primarily due to the absence of external funding, which limited the authors’ ability to extend the study period and increase the number of samples collected. An expanded sampling frame and longer study duration could have provided a more comprehensive understanding of the prevalence and dynamics of multidrug resistance within the hospital setting.

## Conclusion

In this study, resistance rates to selected antibiotics among the clinical isolates obtained ranged from 19.4% to 69.4%, with 73.6% of isolates classified as multidrug-resistant. Biofilm formation was observed in 48.6% of isolates, while phenotypic Extended - Spectrum β-lactamase production was detected in 29.2% (*n* = 21) of the total isolates. Hypermucoviscosity phenotype was identified in 21.1% of *Klebsiella pneumoniae* isolates. Among the genes screened, *bla*_TEM_ was the most prevalent (35.3%), followed by *bla*_CTX−M_ (29.4%), *bla*_SHV_ (23.5%), and *bla*_CMY_ (11.8%). The elevated resistance rates to commonly-used antibiotics, coupled with the high prevalence of MDR phenotypes, underscore the urgent need for public health interventions. Specifically, targeted awareness campaigns are necessary to educate the community on the risks associated with antibiotic misuse, overuse, and unsupervised consumption. Such initiatives are critical to curbing the spread of antibiotic resistance and mitigating its potential adverse impact on environmental and public health.

## Data Availability

“All the data generated or analysed during the execution of this study are included in this published article.”
